# Inter- and intraobserver reliability of the MTM-classification for proximal humeral fractures: A prospective study

**DOI:** 10.1186/1471-2474-9-21

**Published:** 2008-02-17

**Authors:** Christian Bahrs, Hagen Schmal, Erich Lingenfelter, Bernd Rolauffs, Kuno Weise, Klaus Dietz, Peter Helwig

**Affiliations:** 1 BG Trauma Center, Eberhard-Karls-University, Schnarrenbergstr. 95, D-72076 Tuebingen, Germany; 2 Department of Orthopaedic and Trauma Surgery, Albert Ludwig University, Hugstetterstrasse 55, D-79106 Freiburg, Germany; 3 Northland Bone and Joint Institute, 2750 Clay Edwards Dr. 304, Kansas City, MO 64116, USA; 4 Department of Medical Biometry, Eberhard-Karls-University, Westbahnhofstr. 55, D-72070 Tübingen, Germany

## Abstract

**Background:**

A precise modular topographic-morphological (MTM) classification for proximal humeral fractures may address current classification problems. The classification was developed to evaluate whether a very detailed classification exceeding the analysis of fractured parts may be a valuable tool.

**Methods:**

Three observers classified plain radiographs of 22 fractures using both a simple version (fracture displacement, number of parts) and an extensive version (individual topographic fracture type and morphology) of the MTM classification. Kappa-statistics were used to determine reliability.

**Results:**

An acceptable reliability was found for the simple version classifying fracture displacement and fractured main parts. Fair interobserver agreement was found for the extensive version with individual topographic fracture type and morphology.

**Conclusion:**

Although the MTM-classification covers a wide spectrum of fracture types, our results indicate that the precise topographic and morphological description is not delivering reproducible results. Therefore, simplicity in fracture classification may be more useful than extensive approaches, which are not adequately reliable to address current classification problems.

## Background

Proximal humerus fractures have a great variability and complexity. In general only two systems are used for classification: the Neer classification and the classification of the Arbeitsgemeinschaft für Osteosynthesefragen/Association for the Study of Internal Fixation (AO/ASIF) [1-4]. During the last years, a few studies presented fracture types, which were not accurately described with either of the two classification systems [5-7]. Several studies have also shown high rates of inter- and intraobserver variability in both classifications [8-16]. Only the analysis of fractured parts according to Neer and the AO-main-types led to a moderate to substantial reliability. Reliability studies that specifically analysed a very detailed classification (Neer parts and groups, AO – types and groups and subgroups) showed only fair to slight reliability. A low reliability reported in these studies could be a possible reason for discrepancies when comparing outcome studies especially after treatment of complex three-and four-part-fractures [17].

Therefore, new classification systems were introduced during the last years [18,19]. A new system based on the analysis of more than 250 humeral fractures-was developed, which allows a detailed modular classification of proximal humeral fractures condensing Neer and AO with special regard to defined topographic and morphological criteria (MTM-Classification). The current study assessed the reliability of the MTM-classification and compared the results with fractured parts of the proximal humerus according to Neer-classification using plain x-rays. We also wanted to answer the question whether classification of proximal humeral fractures in this very detailed way, exceeding the analysis of fractured parts (according to Neer), may be limited by its reliability.

## Methods

### Presentation of the Classification

The alphanumeric classification consists of a **m**odular **t**opographic and **m**orphologic (MTM-) classification. To facilitate a more precise, reliable and reproducible fracture differentiation, initial radiological assessment, standardized plain x-rays in a.p. and axillary views were mandatory.

### The Topographical Basis

The topographic basis is the division of the proximal humerus into two segments-the articular segment (with C-fractures from C = Caput = Humeral head) and the extraarticular segment. This extraarticular segment (with A-fractures) is further divided into the three subsegments metaphysis (M), Greater tuberosity (G) and Lesser tuberosity (L).

**Figure 1:**
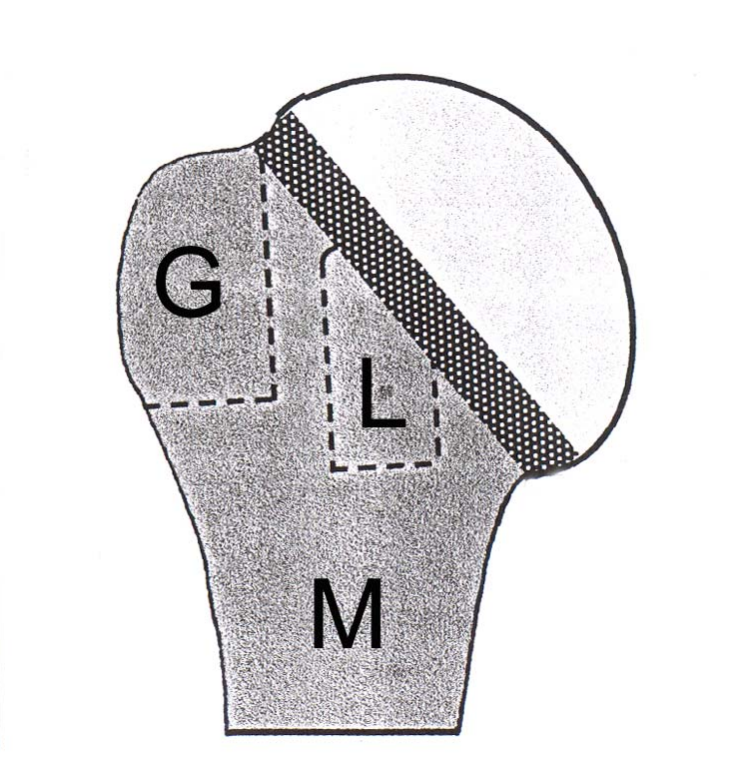
Division of the proximal humerus into the articular segment (humeral head) and the extraarticular segment, further divided in three subsegments: M = metaphysis, G = greater tuberosity and L = lesser tuberosity.

Both the articular and the extraarticular segments are divided by the anatomical neck (see Figure 1).

### The fracture types

**A-Fractures **comprise fractures in the extraarticular segment. Two-part-A fractures are divided in two-part metaphyseal fractures (M) that extend through the surgical neck, two-part greater tuberosity (G) and two-part lesser tuberosity fractures (L). The tuberosity fractures are defined by a complete separation of the tuberosity from the metaphysis and the anatomical neck.

Three-part A fractures (MG and ML fractures) are a metaphyseal fracture (M) with a fracture of one tuberosity (G or L).

**Figure 2:**
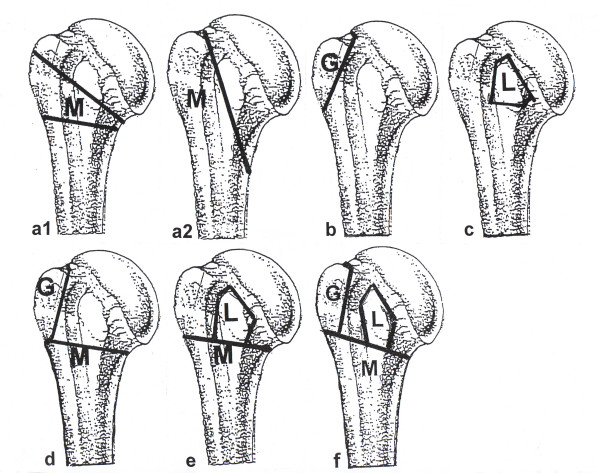
**Schematic illustration of type A fractures**. a1) Typical M fracture = isolated metaphyseal fracture through the surgical neck or as high pertubercular M fracture. a2) Vertical M fracture = isolated metaphyseal fracture coming from the anatomic neck proceeding lateral from the lesser tuberosity to distal medial through the metaphysis. b) G fracture = isolated fracture of the greater tuberosity. c) L fracture = islolated fracture of the lesser tuberosity. d) MG fracture = metaphyseal fracture and fracture of the greater tuberosity. e) ML fracture = metaphyseal fracture and fracture of the lesser tuberosity. f) MT fracture = metaphyseal fracture and fracture of both tuberosities.

Four-part A fractures (MT fracture) are a metaphyseal fracture (M) with a fracture of both tuberosities (G+L = T) (see Figure 2a–f).

### Type B: Incomplete Articular Fractures

Incomplete articular fractures show an incomplete fracture through the anatomic neck and extend into one of the extraarticular subsegments. This type describes a tuberosity or a medial wedge-shaped fragment, which is separated from the metaphysis and remains at the humeral head. Two-part B fractures are divided into three subtypes such as incomplete articular fractures with an medial metaphyseal wedge shaped fragment at the humeral head (MB), an incomplete fracture of the anatomical neck with the greater tuberosity at the humeral head (GB) and an incomplete fracture of the anatomical neck with the lesser tuberosity connected to the humeral head (LB).

The further division of type B fractures depends on the additionally occurring tuberosity fractures. These three-part B fractures are GB fractures with a separate fracture of the lesser tuberosity (GBL), LB fractures with a separate fracture of the greater tuberosity (LBG), MB fractures with a separate fracture of the greater tuberosity (MBG) and MB fractures with a separate fracture of the lesser tuberosity (MBL).

**Figure 3:**
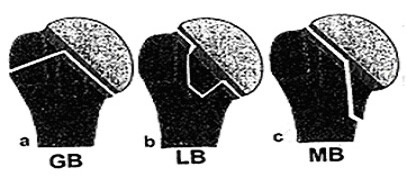
**schematically shows the two-part B fractures**. a) incomplete fracture of the anatomical neck with extension into the greater tuberosity (GB). b) incomplete fracture of the anatomical neck with extension into the lesser tuberosity (LB). c) incomplete articular fractures with an medial metaphyseal wedge shaped fragment at the humeral head (MB).

**Figure 4:**
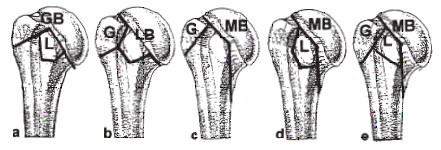
**The further division of type B fractures (Three-part B-fractures) depends on the additionally occurring tuberosity fractures**. a) GBL fractures = incomplete fracture of the anatomical neck with extension into the greater tuberosity (GB) with a fracture of the lesser tuberosity. b) LBG fractures = incomplete fracture of the anatomical neck with extension into the lesser tuberosity (LB) with a fracture of the greater tuberosity. c) MBG fractures = incomplete articular fractures with an medial metaphyseal fragment at the humeral head (MB) with a fracture of the greater tuberosity. d) MBL fractures = incomplete articular fractures with an medial metaphyseal fragment at the humeral head (MB) with a fracture of the lesser tuberosity. e) MBT fractures = 4-Part B fractures appear which are MB fractures in combination with a fracture of both tuberosities (G and L = T) = MBT fractures.

B fractures with four main parts (= 4-part B fractures) are MB fractures in combination with a fracture of both tuberosities (MBT) (see Figure 3 and 4).

### Type C: Complete Articular Fractures

Type C articular fractures completely pass through the anatomic neck, but also may extend through the humeral head, which is completely separated from the extraarticular segment. Type C fractures are divided into five subtypes. Two-part C fractures with just one complete fracture through the anatomic neck are isolated articular fractures (SC).

**Figure 5:**
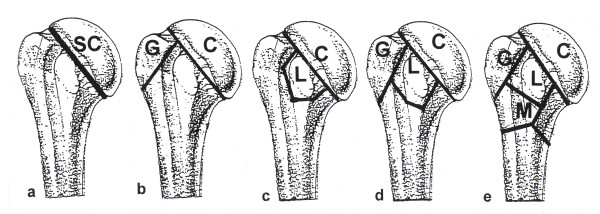
**Schematic illustration of type C fractures**. a) SC fracture = isolated fracture through the anatomic neck. b) CG fracture = complete fracture through the anatomic neck and fracture of the greater tuberosity. c) CL fracture = complete fracture through the anatomic neck and fracture of the lesser tuberosity. d) CT fracture = complete fracture through the anatomic neck and fracture of both tuberosities (G and L = T). e) CTM fracture = complete fracture through the anatomic neck and fracture of both tuberosities and metaphyseal fracture.

Three-part C fractures showing a fracture of the greater tuberosity (CG), with a fracture of the lesser tuberosity (CL). Four-part C fractures show a fracture of both tuberosities fractures (CT). Along with four-part fractures there is often a fracture of the metaphyseal subsegment. These even more complex fractures are called (CTM) (see Figure 5).

### Type D-Fractures (Fracture-Dislocations)

D fractures are fracture-dislocations. D fractures are divided in type C fracture-dislocations (DC), type B fracture-dislocations (DB) and type A fracture-dislocations (DA).

The further classification of DA fractures depends on the fractures in the extraarticular segment: fracture-dislocation with a metaphyseal fracture (DM), anterior fracture-dislocation dislocation and fracture of the greater tuberosity (DG) and posterior fracture-dislocation and fracture of the lesser tuberosity (DL).

### The morphological basis

For morphological analysis, the MTM classification is based on four defined specifications, which are relevant for therapy and prognosis.

**Figure 6:**
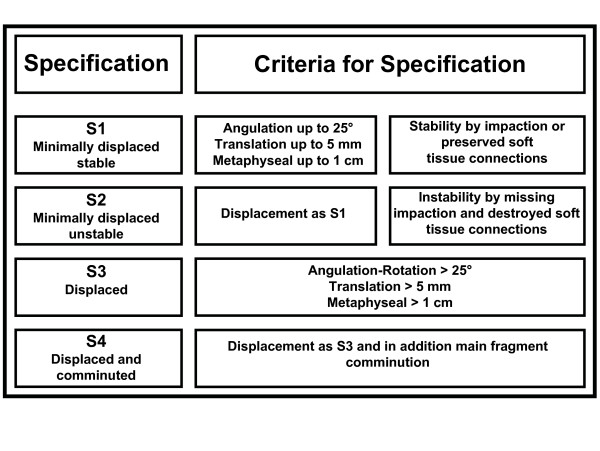
Specification scheme for proximal humeral fractures.

These specifications are organized by increasing fracture severity: minimally displaced and stable (S1), minimally displaced and unstable (S2), displaced (S3), displaced and comminuted (S4). In fractures with several parts, each part has to be classified individually. (see Figure 6).

#### S1: Minimally Displaced and Stable

The minimally displaced fractures (S 1) are defined as fractures with angulations up to 25°, a displacement of the tuberosities and the anatomical neck up to 5 mm, and a metaphyseal fracture displacement of 10 mm. Up to this extent of displacement, a real impairment of shoulder function is not to be expected.

Fracture stability is given if – through impaction of the main parts and preserved soft tissues – mobility between the main parts resulting in further displacement is unlikely. Thus, the fracture position, induced by the trauma, is not changing by careful functional strain of the shoulder.

Therefore, minimally displaced and stable fractures are amenable to nonoperative treatment including early functional exercises. Regardless of fracture type and the number of main fragments, those fractures can be grouped together as S1 fractures since they are almost analogous in treatment and prognosis.

#### S2: Minimally Displaced and Unstable

The displacement of S2 fractures is defined similar to S1 fractures as a displacement of the tuberosities and the anatomical neck up to 5 mm, and a metaphyseal fracture displacement of 10 mm. Those fractures were defined as unstable when fractured parts were not impacted into each other resulting in instability between the fractured parts. Thus, through muscle pull and shoulder mobilization further displacements beyond the initial radiographically diagnosed extent may occur.

##### Assessment of Stable and Unstable Fractures

If the above-defined criteria for stability and instability cannot clearly be applied radiologically, an additional fluoroscopic examination of the fracture is recommended. In this examination, a gentle abduction and rotation is applied in true a.p. view. If mobility can be visualized between fractured parts, the fracture is defined as unstable.

#### S3: Displaced

S3 fractures always show a stronger malalignment and fragment separation, and, thus, the interfragmental soft tissue is ruptured more strongly, mostly induced by a combination of angulatory, rotatory and translatory displacement. Often, a compression mechanism leads to strong displacement with impaction. In displaced C fractures, the blood supply to the humeral head is completely destroyed. In cases with translatory and rotatory displacement of the humeral head, there is no integrity of the medial hinge and capsular and periosteal vessels ascending intraosteally to the humeral head are ruptured[18,20].

#### S4: Displaced and Comminuted

In addition to the displacement, S4 fractures show comminution of the main part complicating the therapeutic procedure and worsening the prognosis. For example displaced head-splitting fractures define the fracture of the humeral head in several single fragments (C4).

### The application of the classification

Due to its modularity, the classification can be applied in several ways depending on application purposes.

The short topographic version allows to classify into main types (A, B, C, D) or into the number of main parts (2, 3, 4) or into a combination of both the main-fracture-type and parts (2-p-A-fracture to 4-p-C-fracture).

A more detailed approach evaluated individual fracture types (M to CTM) or fracture types in combination with the individual specification (M to CTM and S1 to S4).

For example, a stable fracture of the anatomical neck with a displaced fracture of the greater tuberosity could be classified as C1G3.

To allow the comparism with other studies we also classified the fractures according to the determination between minimal-displaced and displaced fractures (S1/S2 to S3/S4).

## Methods

In a prospective study during March 2005 and August 2005 a consecutive series of 22 patients with 22 proximal humeral fractures presented at the BG Trauma Centre of the University of Tuebingen were included into the study. All patients were diagnosed with standardized plain x-rays in a.p. and axillary views with the patient supine using a shoulder splint with at least 60° abduction of the arm[21]. In addition, suspected instability in minimally displaced fractures was assessed using fluoroscopy (n = 4). All x-rays were reviewed by an experienced trauma surgeon, who was not participating in the evaluation to ensure that the x-ray quality of all 22 patients was sufficient for later classification. All x-rays were collected and numerated; patient identification data was rendered anonymously. Then, the x-rays were submitted to three observers. Observer 1 was an experienced orthopaedic surgeon with fellowship training in shoulder surgery. Observer 2 was an experienced trauma surgeon with a special interest in bone and joint orthopaedic surgery. Observer 3 was a fellow for trauma surgery with a special interest in bone and joint surgery. None of the examiners were employed by the hospital, in which the fractures were seen. During routine work of the three observers only the Neer and AO classification were used. None of the observers had any experience with the MTM classification before study onset to exclude the influence of training on reliability.

At the beginning of the study, the MTM classification was provided to the examiners in English and German language. In addition, the first author gave a 15-minute presentation of the MTM-Classification. A goniometer and a pen were given to the examiners. The data acquisition of the three examiners took place independently.

All fractures were classified by the observers according to the following guidelines:

a) Topographic analysis by individual fracture types (M to CTM) with consideration of individual specification (S1 to S4, Figures 6, Table 1).

**Table 1:** shows an overview of the classification of proximal humeral fractures into main parts and fracture types and subtypes.

**Main fracture types**												
	**A**			**B**				**C**		**D**		

**Main Parts**												
**2-part**	M	G	L	GB	LB		MB	SC		DM	DG	DL
**3-part**	MG	ML		GBL	LBG	MBG	MBL	CG	CL			
**4-part**	MT			MBT				CT				

b) Topographical analysis by individual fracture types (M to CTM) (see Table 1).

c) Analysis by a combination of both main fracture types (A, B, C, D) and number of parts (2–4) (see Table 1).

a) Analysis by the 4 main fracture types (A, B, C or D) (Table 1).

d) Analysis by number of main parts (2-part, 3-part, and 4-part according to Neer-classification) (see Table 1).

e) Morphological distinction between minimal-displaced fracture and displaced fracture (S1/S2 or S3/S4) (see Figures 6).

After initial analysis, a second evaluation of the x-rays took place after 4 months. Prior to this part of the study, none of the examiners had knowledge about this second evaluation. The classification was presented again in English and German languages. A routine application of the MTM classification during routine work did not take place. The second evaluation was performed as described above except that radiographs were presented in reverse order. All except one fracture, that was treated conservatively, were stabilized by open reduction and internal fixation with an angular-stable plate osteosynthesis.

### Statistical Methods

Percentage agreement, intraobserver reliability and interobserver reliability were assessed with the JMP-statistical package (Version 6, SAS Campus Drive, Building S, Cary, NC, 27513, USA). For intraobserver reliability and interobserver reliability, the kappa statistic function of the JMP-statistical package was used measuring kappa values (κ) to describe the agreement between observers while correcting for the proportion that may have occurred by chance alone.

A kappa value of 0 represented agreement by chance alone while kappa value of 1 represented a perfect agreement. Kappa values were interpreted using the guidelines proposed by Landis and Koch. Values between 0.81 and 1 indicated excellent or almost perfect, 0.61 and 0.80 substantial, 0.41 and 0.60 moderate, 0.21 and 0.40 fair and 0 and 0.20 slight reliability [22].

## Results

### Intraobserver Reliability

Lowest percentages of agreement were detected for individual fracture type and morphological specification (M to CTM + S1–S4, range 54.5%–68.2%), followed by number of main parts combined with main fracture type (2–4 + A-D, range 68.2%–81.8%) and individual fracture type (M-CTM, range 68.2%–72.7%).

The highest percentage agreement between the observers was found for the parameters of fracture displacement (S1/2 – S3/4, range 90.1%–95.4%), number of main parts (2–4, range 86.4% – 90.1%) and main fracture type (A-D, range 81.8%–90.1%). Statistical analysis showed the lowest kappa values in individual fracture type and morphological specification and the highest intraobserver reliability when fractures where classified according to number of main parts, fracture displacement and main fracture types including number of parts. (See Table 2).

**Table 2:** Kappa values of the intraobserver analysis of the three observers

**Fracture Classification**	**Observer 1 Kappa**	**Observer 2 Kappa**	**Observer 3 Kappa**
**Main-Type (A, B, C, D)**	0.27	0.34	0.71
**Main fragments (2, 3, 4)**	0.73	0.82	0.76
**Main Type + Main fragments (2A – 4C)**	0.41	0.61	0.63
**Fracture Displacement (S1/2 and S3/4)**	0.76	0.46	0.64
**Type of fracture (M-CTM)**	0.48	0.58	0.54
**Type of fracture (M-CTM) & Specification (S1–S4)**	0.39	0.30	0.44

### Interobserver Reliability

The lowest percentage agreement between all observers was found in the assessment of combination of fracture type and individual specification. Followed by the classification according to individual fracture type and main type and parts.

The highest percentage agreement between all observers was found in the assessment of fracture displacement followed by the classification according to main fracture types and fractured parts.

A fair level of agreement was found in the evaluation of individual fracture type in combination with specification and individual fracture type and analysis of main fracture type and main parts. A moderate to substantial level of agreement was found in the evaluation of fracture displacement and number of main parts. (See Table 3).

**Table 3:** Kappa values of the interobserver analysis of the three observers

	**Observer 1&3**		**Observer 1&2**		**Observer 2&3**		**Observer 1–3**	
	
	**%**	**Kappa**	**%**	**Kappa**	**%**	**Kappa**	**%**	**Kappa**
**Main-Type**	77.3	0.19	84.1	0.39	79.5	0.32	80.3	0.30
**Main fragments**	68.2	0.41	79.5	0.60	75.0	0.55	74.2	0.52
**Main Type & Main fragments**	56.8	0.25	65.9	0.39	61.3	0.36	61.3	0.33
**Fracture Displacement**	95.4	0.73	93.2	0.63	97.7	0.85	95.4	0.74
**Type of fracture**	54.5	0.31	61.3	0.40	56.8	0.37	57.5	0.36
**Type of fracture & specification**	63.6	0.31	56.8	0.45	47.7	0.4	49.2	0.39

## Discussion

The MTM-Classification was developed to advance understanding of proximal humeral fractures. Especially by introduction of incomplete articular fractures (B-fractures) a topographic classification could by possible. By adding morphological aspects and the possibility of a modular application of the system a precise evaluation of almost all fractures may be possible as well. However, the classification of proximal humeral fractures with the MTM-systems results in various differences in reliability depending on the short or extensive version of application. Analyzing the short version of the classification, the interobserver analysis resulted in moderate kappa values in the category 'main parts' (according to Neer-parts) and substantial kappa values for analyzing 'fracture displacement'. Those results were in accordance with the literature[8,23]. Analyzing the extensive version of the MTM-Classification like evaluation of the main or individual fracture type with inclusion of the specification, only fair results can be expected.

At present the classifications by Neer and the AO for proximal humeral fractures are widely accepted and commonly used although both classifications have received some criticism during the last years [1,3]. Neither of the two systems achieved the general requirement of classifications to adequately cover the complete spectrum of all possible fracture patterns. The Neer-system does not include 4-part valgus impacted fractures (covered by the AO-Classification as C2.1 or C2.2)[24]. Furthermore, the Neer-classification describes a large and rather inhomogeneous group (one-part fractures/minimal-displacement) that consists of all fractures that are angulated less than 45° and displaced less than 1 cm, independently of the number of fractured parts. More so, 3 and 4-part fractures are divided by Neer in types of lesser tuberosity and greater tuberosity fractures. In these complex fractures, it remains unclear whether a fracture runs through the anatomical or the surgical neck. Furthermore, the AO classification includes three types (A, B and C) each divided into 3 groups and 9 subgroups. For further subgroup description, 41 qualifications were used. Despite this detailed approach, complex injuries such as fractures of the anatomical neck with both tuberosities (4-part fractures according to Neer) or isolated lesser tuberosity fractures are inadequately defined.

New classifications systems such as the MTM classification were developed to address these critical points[18,19]. As a main prerequisite for correct topographic and morphologic fracture assessment, standardized and good-quality anteroposterior and axillary radiographs are required. Tamai published a series of 22 3 and 4-part fractures. He compared plain x-rays in anteroposterior and scapula lateral views with the intraoperative gross anatomy. Eight cases (36.4%) could neither be classified by Neer nor by the AO classification system. The authors concluded that diagnostics in complex fracture situations are difficult and that the classifications used were not as accurate as one would hope. Furthermore, Tamai attributed present difficulties in the choice of correct therapeutic methods to an inadequate fracture classification system[7].

The MTM classification advanced available classifications by including the fracture model of Codman, the Neer analysis of fractured parts and the AO differentiation of fracture height, allowing for the description of almost all possible fracture types [1,3,25].

Published reliability studies differ in observer experience and diagnostic imaging methods. In addition, partly simplified Neer and AO classifications were used, leading only to limited study comparisons. (see Table 4).

**Table 4:** Selected results of reliability studies of classifications for proximal humeral fractures

**Study group**	**Classification**	**No. of categories + (Image modality)**	**Overall or mean (range) kappa values for intraobserver reliability**	**Overall % (range) of observer agreement**	**Overall or mean (range) kappa values for intraobserver reliability**
**Kristiansen et al. 1988 [16]**	Neer	5 (x-ray)		(24–59)	0.30 (0.07–0.48)
		3 (x-ray)		(49–75)	0.29 (0.03–0.47)
**Sidor et al. 1993 [10]**	Neer	16 (x-ray)	0.66 (0.50–0.83)	32	0.48 (0.43–0.58)
**Siebenrock et al. 1993 [11]**	Neer	4 (x-ray)	0.60 (0.46–0.71)	26	0.40 (0.25–0.51)
	AO Types	3 (x-ray)	0.58 (0.54–0.66)	38	0.53 (0.50–0.58)
	AO Groups	9 (x-ray)	0.48 (0.43–0.54)	15	0.42 (0.36–0.49)
**Brien et al. 1995 [15]**	Neer	3 (x-ray)		(57–71)	(0.37–0.75)
**Bernstein et al. 1996 [8]**	Neer	16 (x-ray)	0.64		0.52
		16 (CT)	0.68		0.56
		16 (CT+x-ray)	0.72		0.50
**Sjöden et al. 1997 [12]**	AO Groups	9 (x-ray)	(0.16–0.60)		0.31
	Neer	6 (x-ray)	(0.20–0.85)		0.42
**Sjöden et al. 1999 [13]**	AO Groups	9 (x-ray)	(0.29–0.74)		0.32
	Neer	6 (x-ray)	(0.27–0.73)		0.44
**Sharder et al. 2005 [23]**	Neer	16 (x-ray)	(0.19–0.73)	42	0.42
**Mora Guiz et al. 2006 [14]**	Neer	4 (x-ray)	0.27		0.35
		4 (x-ray+CT)	0.30		0.44
**Brorson et al. 2002 [30]**	Neer	6 (x-ray/initial)			0.27
		6 (x-ray after training)			0.62

Also, comparisons of kappa values from different studies may be problematic, as kappa values change with the prevalence of the diagnosis[26]. Despite these problematic aspects, the author's selected a few studies with comparable methods for discussion.

Several authors assess the intraobserver reliability and interobserver reproducibility. Using conventional radiography and in some studies additional CT-scans, they found a fair to substantial mean intraobserver agreement as well as a fair to substantial mean interobserver agreement for the Neer and for the AO classification. (see Table 4).

The present study used standardized plain x-rays, enlisted experienced trauma surgeons and used the whole range of the classification spectrum. This approach led to an average percentage agreement of the three observers of 49.2% with a fair kappa value of 0.39 for fracture-analysis under consideration of individual fracture type and specification.

Siebenrock and Gerber also stated that the AO classification with three main types (A-C) resulted in better kappa values than the modified Neer classification with four choices of fracture types [11]. These findings were confirmed by our study, in which we found acceptable to good agreement in 2 to 3 groups for determination of fracture displacement or fractured main parts. On the other hand, Sidor found that a simplification of the Neer classification from 16 into 6 categories did not result in significant changes of interobserver reliability[10]. However, a 2-choice question, for example about therapeutic management or (as in the present study) about fracture displacement led to perfect and good reliability, indicating a dependency of interobserver reliability and the number of choices the observer has to select[8,23].

Sidor also discussed that a differentiation of single fragments due to multiple fractures lines is difficult. He also stresses the importance of a high-quality radiological diagnostics, which makes an overlapping free presentation for the fractured region possible to avoid any classification restriction. For an excellent classification of proximal humeral fractures, a perfect radiological visualization of the fractured region is mandatory[10]. In accordance with other experts, it is possible to increase the information with an axillary view in terms of direction and dimension of fracture dislocation, humeral head involvement, and the degree of displacement of tuberosity fractures, especially considering fractures of the lesser tuberosity [27-29].

Brorson and Shrader analyzed the importance of training in a specific classification system. They could show that kappa values for interobserver reliability were significantly improved from fair to substantial after observers had received a classification training[23,30,31].

Since the training in a specific classification system improves reliability, one weakness of the current study was, that the MTM-classification was not used in daily clinical practice. With sufficient training, the reliability of this classification could be higher.

## Conclusion

In summary, some complex fracture types are inadequately defined by classification systems such as the present Neer or AO classification. To allow a precise topographic and morphological description, the MTM classification was developed for a better understanding of individual fractures and to address the question whether a very detailed classification of proximal humeral fractures may be limited by its reliability. Unfortunately, the very detailed classification approach led only to fair or unacceptable results and is not helpful to improve reliability.

We concluded that a detailed classification exceeding the part analysis of Neer is not a practical approach to address current problems in classification systems regarding proximal humerus fractures.

For future projects an evaluation of ongoing developments of diagnostic imaging-technology like the CT and here specifically multiplanar visualisation of the fracture in thin-cut technique and 3 D visualisation should be undertaken.

## Pre-publication history

The pre-publication history for this paper can be accessed here:


